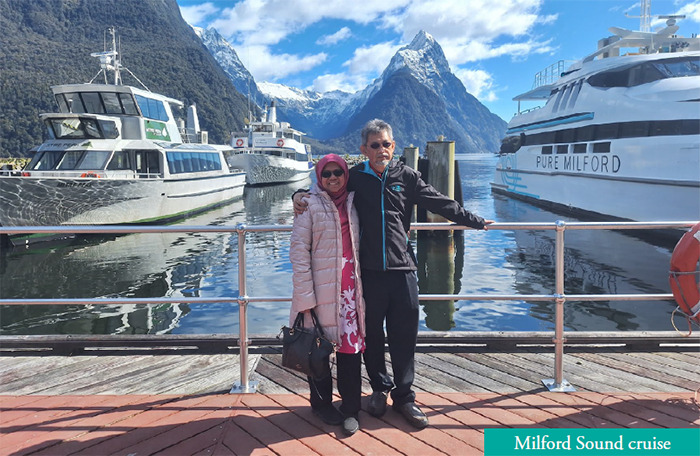# Story of a diving mouse and a driving man

**DOI:** 10.51866/mol.1061

**Published:** 2026-02-12

**Authors:** Ruzilawati Abu Bakar, Imran Ahmad

**Affiliations:** 1 Department of Pharmacology, School of Medical Sciences, Universiti Sains Malaysia, Kubang Kerian, Kelantan, Malaysia.; 2 Department of Family Medicine, School of Medical Sciences, Universiti Sains Malaysia, Kubang Kerian, Kelantan, Malaysia.

**Keywords:** Mice, Diving, Adaptation, Survival

Queenstown Research Week is an annual event held in Queenstown, New Zealand. This year, it was relocated to Christchurch to accommodate a larger venue. My wife and I were there from the 3^rd^ to the 5^th^ of September 2025, attending the event for the first time. I presented a paper on the burden of carers of patients with a diabetic foot. My wife presented her poster on genetic expression in chronic hepatitis C.

What we found most interesting was listening to the plenary lecture by one of the speakers. He was talking about the genetic component of obesity in children of mothers with postpartum depression using an animal model. In assessing depression in mice, they employed the forced swim test. Mice do not like water and would keep swimming, trying to escape. Mice with depression would spend more time immobile and eventually sink.

During testing, the authors manipulated certain enzymes working in the brains of the mice. They were surprised by an accidental finding. Deleting certain enzymes caused the mice to be adventurous, explorative and brave. These mice were not afraid of water; instead, they dived to the bottom and resurfaced. This had never been documented in laboratories before.

Following the conference, we embarked on the second part of our trip. We drove to the south of the island, stopping at Tekapo, Mt Cook and Wanaka before finally arriving in Queenstown at night. The ultimate destination was Milford Sound the following morning.

We started our journey just before 6 a.m., aiming to reach Milford Sound by 10 a.m., as our cruise was scheduled to leave by 10:30 a.m. I switched on Google Maps on my smartphone. The information displayed was depressing enough. It showed the route we were supposed to take, a 4-h drive. However, a portion of the road, a few kilometres towards the end, was closed because of an avalanche, and there were no alternative roads. Our hope of visiting Milford Sound was dashed, but we decided to continue with the trip and enjoy the scenery, driving slowly instead. When we reached Te Anau town, roughly halfway through, we received information that the road had been re-opened. We then decided to continue driving to Milford Sound.

We reached Milford Sound at almost 12 noon and had certainly missed our boat. However, we were delighted when the lady at the counter informed us that we could join the next scheduled trip using the same tickets. We boarded the cruise at 12:45 p.m. The 1-h return trip brought us from the jetty to the edge of the open sea. We were cruising between snow-capped mountains interspersed with waterfalls. The pod of dolphins dancing on the water surface and the lone furry seal basking on her favourite rock added to the excitement.

The road closure was a blessing in disguise. It allowed us to travel unhurried, making many stops to enjoy the scenery. Perseverance is key. Just as the mouse dives to explore for survival, I drive to explore for revival.

**Figure:**
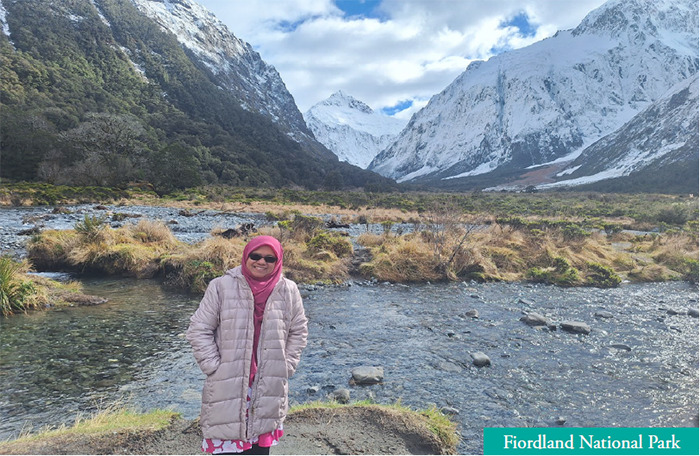


**Figure:**